# Virus‐induced gene silencing database for phenomics and functional genomics in *Nicotiana benthamiana*

**DOI:** 10.1002/pld3.55

**Published:** 2018-04-23

**Authors:** Muthappa Senthil‐Kumar, Mingyi Wang, Junil Chang, Venkategowda Ramegowda, Olga del Pozo, Yule Liu, Vanthana Doraiswamy, Hee‐Kyung Lee, Choong‐Min Ryu, Keri Wang, Ping Xu, Joyce Van Eck, Suma Chakravarthy, Savithramma P. Dinesh‐Kumar, Gregory B. Martin, Kirankumar S. Mysore

**Affiliations:** ^1^ Noble Research Institute Ardmore Oklahoma; ^2^ National Institute of Plant Genome Research New Delhi India; ^3^ Boyce Thompson Institute for Plant Research Ithaca New York; ^4^ Department of Plant Biology and the Genome Center College of Biological Sciences University of California Davis California; ^5^ Section of Plant Pathology and Plant‐Microbe Biology School of Integrative Plant Science Cornell University Ithaca New York; ^6^ Present address: Instituto de Bioquímica Vegetal y Fotosíntesis Universidad de Sevilla/Consejo Superior de Investigaciones Científicas Avda Américo Vespucio 49 41092 Sevilla Spain; ^7^ Present address: Molecular Phytobacteriology Laboratory KRIBB Daejeon 305‐806 South Korea

**Keywords:** functional genomics, gene silencing, *Nicotiana benthamiana*, tomato, virus‐induced gene silencing

## Abstract

Virus‐induced gene silencing (VIGS) is an important forward and reverse genetics method for the study of gene function in many plant species, especially *Nicotiana benthamiana*. However, despite the widespread use of VIGS, a searchable database compiling the phenotypes observed with this method is lacking. Such a database would allow researchers to know the phenotype associated with the silencing of a large number of individual genes without experimentation. We have developed a VIGS phenomics and functional genomics database (VPGD) that has DNA sequence information derived from over 4,000 *N. benthamiana* VIGS clones along with the associated silencing phenotype for approximately 1,300 genes. The VPGD has a built‐in BLAST search feature that provides silencing phenotype information of specific genes. In addition, a keyword‐based search function could be used to find a specific phenotype of interest with the corresponding gene, including its Gene Ontology descriptions. Query gene sequences from other plant species that have not been used for VIGS can also be searched for their homologs and silencing phenotype in *N. benthamiana*. VPGD is useful for identifying gene function not only in *N. benthamiana* but also in related Solanaceae plants such as tomato and potato. The database is accessible at http://vigs.noble.org.

## 1 INTRODUCTION

*Nicotiana benthamiana*, a member of the Solanaceae family, is a model plant species that is widely used for studying host–pathogen interactions and for transient protein expression to examine protein function, subcellular protein localization, and protein–protein interactions (Anand et al., 2007; Chakravarthy, Velásquez, Ekengren, Collmer, & Martin, 2010; Gilbert & Wolpert, 2013; Goodin, Zaitlin, Naidu, & Lommel, 2008; Kaundal et al., 2017; Lee et al., 2017; Liu et al., 2004; Rojas et al., 2012; Wang et al., 2012). In addition to these uses, *N. benthamiana* is also an attractive model to study the function of genes involved in abiotic stress responses, plant development, and metabolism (Chakravarthy et al., 2010; Gas‐Pascual, Berna, Bach, & Schaller, 2014; Jones, Keining, Eamens, & Vaistij, 2006; Liu et al., 2004; Ramegowda, Senthil‐kumar, Udayakumar, & Mysore, 2013; Senthil‐Kumar, Lee, & Mysore, 2013). Although genome sequence and transcriptome data for *N. benthamiana* and several other Solanaceae plants are available (Bombarely et al., 2012; http://sgn.cornell.edu; https://btiscience.org/our-research/research-facilities/nicotiana-benthamiana/), the function of most genes is not known. One of the main reasons for this lacuna is the absence of a large collection of mutants for these Solanaceae species. Although now it is possible to generate targeted mutations using genome editing technologies (Belhaj, Chaparro‐Garcia, Kamoun, & Nekrasov, 2013; Jacobs, Zhang, Patel, & Martin, 2017), these methods require the development of stable transformants which is both resource‐ and time‐consuming. Therefore, virus‐induced gene silencing (VIGS) continues to offer an attractive alternative strategy to determine gene function in Solanaceae plants (Baulcombe, 1999; Burch‐Smith, Anderson, Martin, & Dinesh‐Kumar, 2004; Lacomme, 2011; Robertson, 2004; Senthil‐Kumar & Mysore, 2011a). VIGS exploits the innate plant defense system against viral proliferation and movement. The dsRNA intermediates are recognized by the components of post‐transcriptional gene silencing machinery and provoke targeted degradation of the virus RNA. VIGS is simple, rapid, and silencing phenotypes can be observed within few weeks (Nekrasov, Staskawicz, Weigel, Jones, & Kamoun, 2013). Efficient VIGS for achieving gene silencing for the entire plant growth duration has been reported in *N. benthamiana* (Senthil‐Kumar & Mysore, 2011a,b, 2014). The function of genes from other solanaceous plants can also be studied in *N. benthamiana* with VIGS using heterologous gene sequences (Dong, Burch‐Smith, Liu, Mamillapalli, & Dinesh‐Kumar, 2007; Senthil‐Kumar, Hema, et al., 2007). Finally, VIGS is a powerful tool for “fast‐forward” phenomics and functional genomics screens (Baulcombe, 1999; Burch‐Smith et al., 2004; Senthil‐Kumar & Mysore, 2011a) which have complemented the lack of genetic resources for determining gene function in *N. benthamiana* (Gilbert & Wolpert, 2013; Rojas et al., 2012).

*Tobacco rattle virus* (TRV)‐based VIGS vectors are popularly used for VIGS in solanaceous plants. TRV has two genomes, TRV1 and TRV2, and both the genomes are required for viral replication and movement (Liu, Schiff, Marathe, & Dinesh‐Kumar, 2002; Senthil‐Kumar & Mysore, 2014). The TRV‐VIGS‐based fast‐forward genetics approach has been widely used in *N. benthamiana* to identify plant genes involved in disease resistance, *Agrobacterium*‐mediated transformation, flower development, and coronatine/victorin‐induced cell death (Anand et al., 2007; Chakravarthy et al., 2010; del Pozo, Pedley, & Martin, 2004; Gilbert & Wolpert, 2013; Kaundal et al., 2017; Lee et al., 2017; Lu et al., 2003; Rojas et al., 2012; Senthil‐Kumar et al., 2013; Wangdi et al., 2010). These studies have generated phenotypic data for a large number of gene‐silenced plants. However, these data are not available in a single platform for researchers. As a first step toward integrating these data, we developed a “VIGS phenomics and functional genomics database” (VPGD) that compiles data from the silencing of 4,117 *N. benthamiana* genes. Approximately 1,000 of these genes produced a visible phenotype when silenced and is described in our database. These data will enable researchers to determine phenotypes associated with individual gene knockdowns without performing an experiment. We expect that the VPGD will be a useful resource for a wide range of researchers working with *N. benthamiana* and other economically important solanaceous plants such as tomato, potato, and pepper.

## 2 EXPERIMENTAL PROCEDURES

### 2.1 Plant growth and environmental conditions

*Nicotiana benthamiana* seeds were germinated on trays containing Professional Blend soil (SUN GRO Horticulture Distribution Inc. Bellevue, WA) in a growth chamber. Three‐week‐old *N. benthamiana* seedlings were transplanted to four‐inch pots containing the same soil as above and grown in glasshouse under the following conditions: 20 ± 2°C, 70% humidity, and 16‐hr photoperiod at 50–100 μE s^−1^ m^−2^ light intensity. Two to three days after transplanting, the plants were used for VIGS.

### 2.2 VIGS

cDNA libraries were made from *N. benthamiana* leaf tissue treated with biotic and abiotic elicitors or TMV and cloned into the pTRV2‐based gateway vector and transformed into *A. tumefaciens* GV2260 as previously described (Senthil‐Kumar et al., 2013).

The cDNA library in 96‐well plates was transferred to large plates containing solid LB agar medium and grown for 2 days at 28°C. Bacterial cells of *A. tumefaciens* GV2260 containing the pTRV1 vector were pelleted from overnight cultures, washed twice, resuspended in MES buffer (10 mM MgCl_2_, 10 mM MES) with the optical density at OD_600_ = 1.0, and were infiltrated using a needle‐less syringe into the lower leaves of *N. benthamiana* plants. The individual pTRV2 derivative cDNA clones were grown on LB agar medium plates for 2 days and directly picked using a toothpick and pricked on the leaf area infiltrated with the pTRV1. Plants were kept in the glasshouse (20 ± 2°C; 70% relative humidity; and 16‐hr photoperiod at 50 to 100 μE s^−1^ m^−2^). Two to four plants were inoculated per clone for screening. Plants infected with TRV::*GFP* were considered as virus vector control (Senthil‐Kumar & Mysore, 2011b).

### 2.3 Recording phenotype information

Phenotype information was recorded between 2 and 4 weeks after TRV inoculation. During this period, all visible phenotypic symptoms were systematically recorded at several day intervals and compared with the vector‐only inoculated plants. Composite information obtained from these observations that showed consistency in phenotypes throughout development was finalized, and photographs were taken. VIGS for the selected clones showing a phenotype of interest was repeated to confirm the response. This second‐level screening was carried out to eliminate false positives from the first screen.

## 3 RESULTS

### 3.1 VIGS database

**Figure 1:**
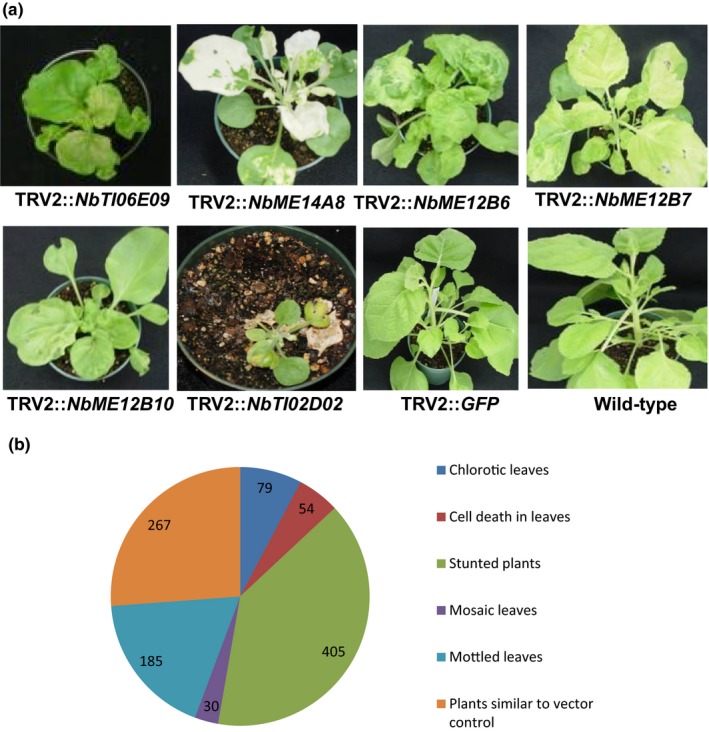
Representative phenotypes presented in the VIGS database and distribution of silenced plants in phenotypic categories. Silencing of NbME and NbTI library genes by VIGS resulted in various phenotypes in *Nicotiana benthamiana*. Representative photographs of some of the most common phenotypes are presented. Plants were photographed approximately 3 weeks after inoculation with the TRV‐VIGS vector (a). Approximately 1,300 plants were individually scored for the visual phenotypes under the mentioned categories. The chart represents the distribution of silencing phenotypes of genes from the libraries (b). Phenotype descriptions: NbTI06E09 silencing shows severely stunted plants, crinkled leaves, reduced apical growth, and severe cell death on top leaves; NbME14A8 silencing shows stunted, bushy plants, and albino green leaves; NbME12B6 silencing shows stunted, bushy plants, green‐white mottled, crinkled leaves, and spotted cell death on leaves; NbME12B7 silencing shows moderately stunted plants and yellow leaves; NbME12B10 silencing shows severely stunted plants, thick, and mosaic leaves; NbTI02D02 silencing shows cell death. Four replicates were carried out for each experiment, and two independent experiments were performed

*Nicotiana benthamiana* mixed elicitor (NbME) (Anand et al., 2007; del Pozo et al., 2004) and *Tobacco mosaic virus* (TMV)‐induced (NbTI) normalized cDNA libraries were cloned into a TRV2‐VIGS vector (Liu et al., 2002; Senthil‐Kumar & Mysore, 2014) and transformed into *Agrobacterium tumefaciens* strain GV2260. The library was arrayed in 96‐well plates such that each well contains a single cDNA clone. To initiate VIGS, a single *Agrobacterium* colony derived from each well was selected using a toothpick and inoculated into the same leaf area where an *Agrobacterium* culture carrying pTRV1 was syringe infiltrated in 3‐week‐old *N. benthamiana* plants (Senthil‐Kumar et al., 2013). TRV1 encodes for an RNA‐dependent RNA polymerase, movement protein, and 1 KDa protein. The description of phenotypes was recorded, and photographs of plants showing visible phenotypes were taken between 2 and 4 weeks postinoculation. Phenotype descriptions and photographs were incorporated into the VPGD. Gene‐silenced plant phenotypes varied and included visible phenotypes such as leaf chlorosis, spotted cell death, stunted growth, leaf curling, leaf crinkling, and leaf mottling. Representative photographs of these phenotypes are shown in Figure 1a. Phenotypes of ~30% of the gene‐silenced plants were no different from the empty TRV vector control (Figure 1b). The most common phenotype recorded in the database was stunted growth (Figure 1b).

**Figure 2:**
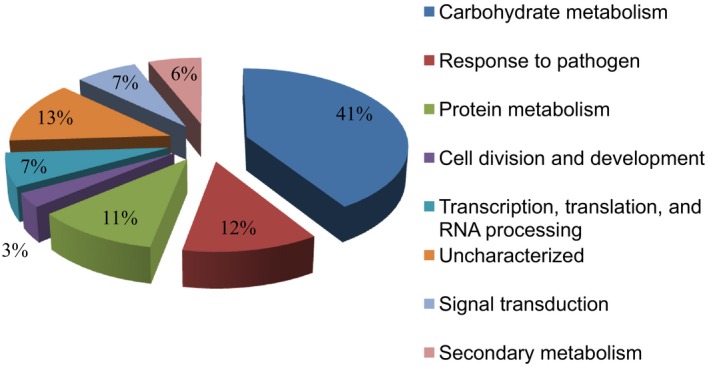
Functional categories of ESTs in the database. Nucleotide sequences in the VIGS database were annotated, and the gene function(s) was identified by BLAST analysis. The pie chart shows the representation of the sequences under different functional categories

To determine the identity of the cDNA sequence in each TRV2 clone, NbME and NbTI cDNA libraries were sequenced by the Sanger method. cDNA inserts from the TRV2 clones were PCR amplified from each well using vector‐specific primers and electrophoresed on an agarose gel to ensure a single band would represent a single colony. Only the colonies showing a single insert were selected for plasmid purification and sequenced using vector‐specific primers. Resulting sequences were processed to remove vector sequences and submitted as EST sequences to NCBI and incorporated into the VPGD. In total, we added 2,779 and 1,332 ESTs from the NbME and NbTI libraries, respectively. Further, these sequences were annotated and classified according to predicted gene function (Figure 2). Each gene sequence, its annotation, and phenotype were matched and were incorporated into VPGD.

**Figure 3:**
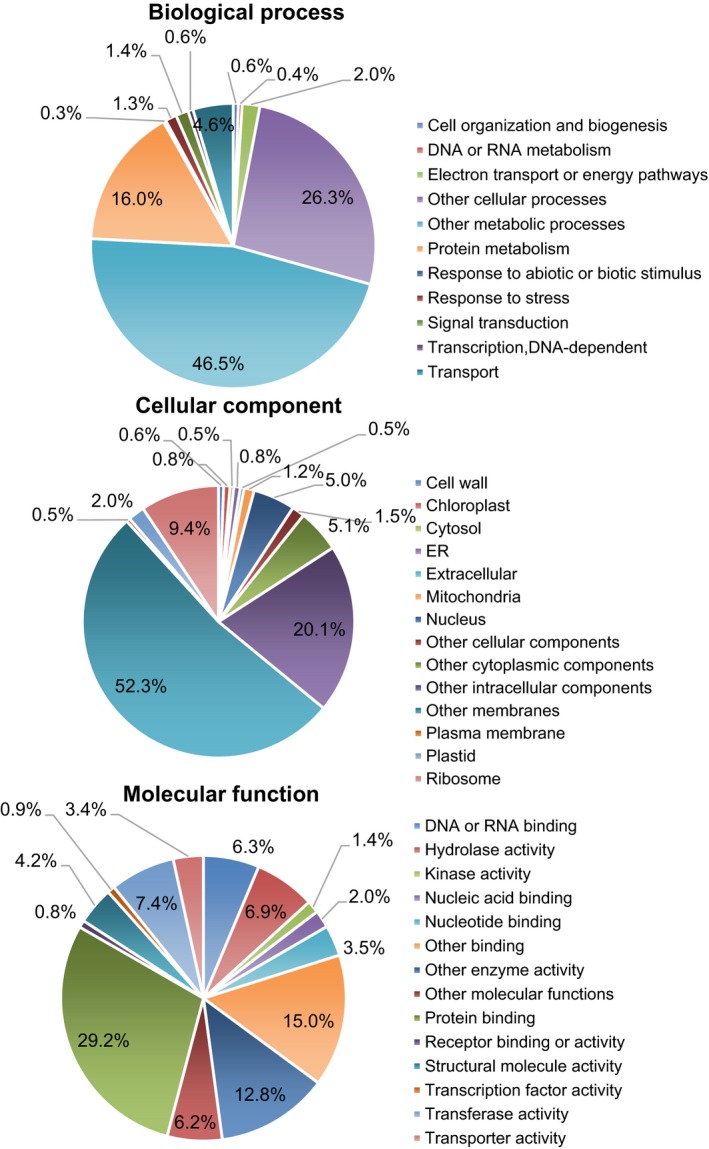
Summary of the gene ontology analysis for the *Nicotiana benthamiana* ESTs. The distribution of gene ontology (GO) terms in three GO categories (biological process, cellular component, and molecular function) is shown in the pie chart. The number of GO terms is counted according to the heterologous BLAST search from Arabidopsis. The charts presented are based on information from tomato. All 4,117 EST sequences were used for the GO analysis

To annotate the EST sequences, we used BLASTX and compared them against three databases (i.e., the Arabidopsis protein sequence, tomato protein sequence, and NCBI protein sequence from all plant species). The top hits with “*e*” values lower than 1*e* ^−10^ were kept, and the related Gene Ontology (GO) term and function description (Jain et al., 2013) were used for annotation. For each GO ID, the VPGD provides the related GO term and associated annotation information. GO terms are widely used to understand the biological significance of genes. We used Arabidopsis and tomato annotations for categorization of ESTs based on GO terms such as molecular function, biological process, and cellular component. The GO terms categories associated with *N. benthamiana* ESTs derived by homologies to tomato are shown in Figure 3. In biological processes, “cell organization and biogenesis,” “other cellular processes,” and “protein metabolism” were the most dominant terms with 47%, 26%, and 16% of ESTs, respectively. In the cellular component category, “other membranes” and “other intracellular components” contributed to 70% of the annotations. In the molecular function category, “protein binding” (29%), “other binding” (15%), and “other enzyme activity” (13%) were the most represented classes.

#### 3.1.1 Site usage

The home page tab provides information about the utility of the database and brief information about the contents of the web site. Contact information and the articles related to the VIGS clones and protocols are also provided. Under the “VIGS Database” tab is a complete list of clones from NbTI and NbME libraries and their associated phenomic information. This information is accessible by selecting the appropriate library using a drop‐down menu. Complete information about the libraries and the VIGS screen used for obtaining the phenotype are accessed by clicking the “About the VIGS library” link in the VIGS Database tab. A keyword search based on the sequence ID, phenotype, and gene name is one of the accessible features. Upon clicking the clone number, its sequence, annotation based on GO terms, the silencing phenotype description, transcript expression data (when available), and a photograph (when available) are displayed and are downloadable. In addition, users can further analyze the sequence to find off‐target genes, efficient siRNAs, and use various other tools by clicking appropriate links at the end of the sequence (http://vigs.solgenomics.net/).

Importantly, the clones listed in this database are available for distribution to researchers around the world, and this information is provided under the “Materials Request” tab. Also provided is information on biosafety and permit requirements. This database is BLAST search enabled. Users can input their query sequence and look for the information related to genomics and phenomics. As *N. benthamiana* is a close relative of many crop plants belonging to the Solanaceae family, sequences from these plant genomes can be used to find their respective homologs in *N. benthamiana* and thereby predict their silencing phenotype information. The database is designed with an option to allow easy addition of more information. For example, phenomic information related to other plant species and other VIGS vectors can be added under the existing architecture. Going forward, VIGS data from other research groups will be added as it becomes available.

### 3.2 Mode of data collection, deposition, and database construction

**Figure 4:**
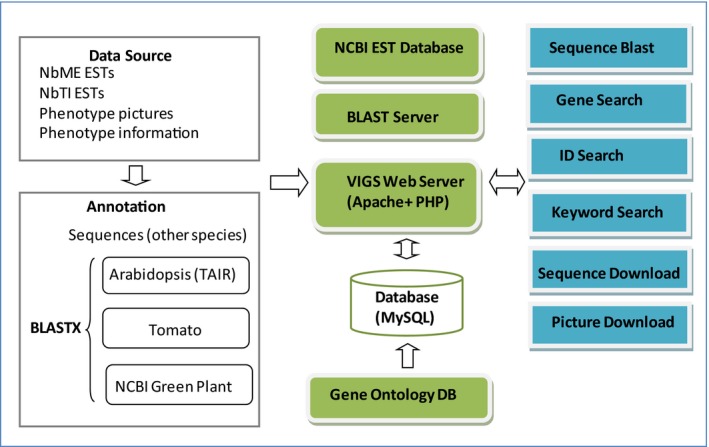
Pipeline of the VIGS database construction. The framework for the VIGS database along with its content organization is presented. The database was constructed based on three inputs namely two EST library sequences, photographs of visual phenotypes, and phenotype descriptions. These inputs were processed as indicated in the middle panel. Specifically, the nucleotide sequences were annotated and a BLAST search was constructed. Phenotype information was organized and presented in a searchable format. Users are able to perform the functions as depicted in the right‐most panel

The VPGD web site was constructed using PHP script, an Apache server combined with the MySQL database on a Linux system. In this web site, the sequences and related silencing phenotype information of ESTs or genes derived from two cDNA *N. benthamiana* libraries (NbTI and NbME) were collected, annotated, and finally imported to the MySQL database (Figure 4).

#### 3.2.1 Sequence acquisition

In order to annotate function of EST sequences, we used BLASTX to compare protein sequences from related plant species. The Arabidopsis sequences were downloaded from the TAIR web site (www.arabidopsis.org). The tomato and draft *N. benthamiana* genome sequences were downloaded from the Sol Genomics Network (http://solgenomics.net). To get all the NCBI plant protein sequences, we also used NCBI Entrez protein search and set “green plants” as the “Taxonomic Groups” filter. The function annotation information of those top hits from related databases and the GO terms from annotated *Arabidopsis* and tomato sequences were extracted and imported into the database.

To provide more details for the GO terms, we also downloaded the full GO annotation database from http://geneontology.org and integrated it into the VPGD. After compiling all the data, the detailed annotation information of each EST including phenotype descriptions, gene function annotations, GO IDs, and their related GO annotation information was added to the database. We also set up a BLAST server and used NbME and NbTI EST sequences as the target database. Users can search the ESTs of interest using this BLAST server.

#### 3.2.2 Accessing VPGD

The VIGS phenomics and functional genomics database is accessible at http://vigs.noble.org or http://bti.cornell.edu/research/projects/nicotiana-benthamiana. Users can find the cDNA sequences, gene silencing plant phenotype description, transcript downregulation (RT‐qPCR), and pictures by browsing through the gene ID. Keyword search options facilitate retrieval of this information based on phenotype description or gene name. From the time of the first database release on October 2014 until December 2017, the VIGS database has had ~66,200 visits.

## 4 DISCUSSION

**Figure 5:**
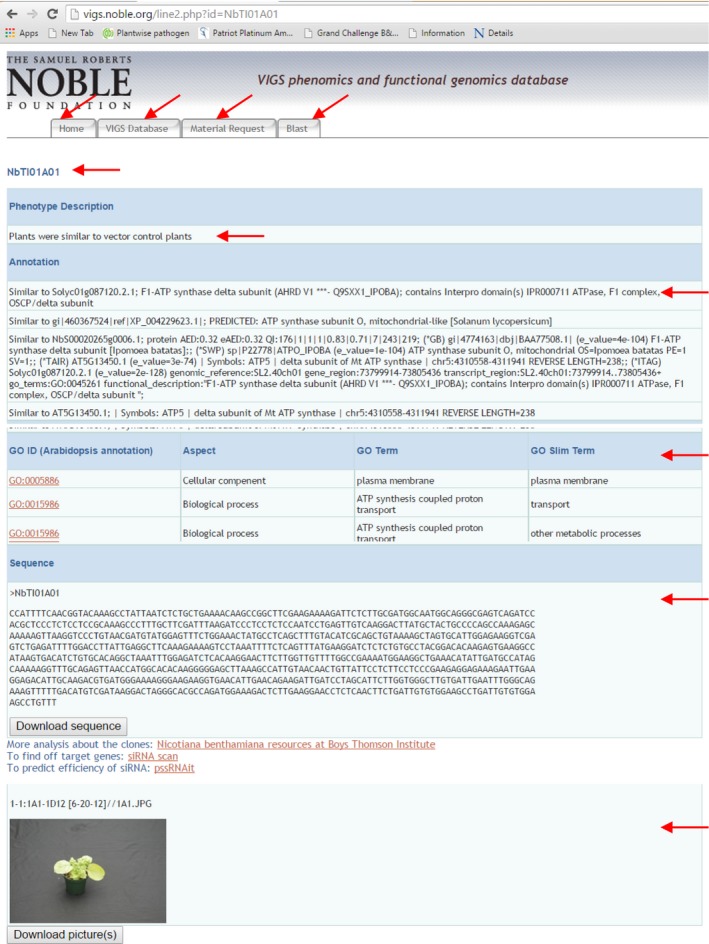
Screenshot of few tabs from the database and description of contents. This screenshot is taken from the VPGD web site. It has four tabs namely, Home, VIGS Database, Material Request, and BLAST. The Home tab contains the basic description of the database including the background and relevant literature. The VIGS Database page displays all information related to the clone upon clicking the clone name or NCBI ID or keyword search. The display includes silencing phenotype description, gene name annotation, GO terms, EST sequence, and photograph of silencing phenotype. The Material Request tab provides details of construct availability and biosafety information. The BLAST tab has a built‐in BLAST page. Query sequence can be used to search for homologs in the database, and this will show all the information available for the desired clone. The red arrows indicate key information provided in the database, and this is to guide the readers to notice the description in the legend

The VPGD was specifically designed to facilitate the interaction between the user and the software. For example, the scheme of information presented in all pages is consistent and there is simple navigation to each category within the database. The results of gene silencing in *N. benthamiana* for a large number of genes can be searched using different methods, including nucleotide sequence, phenotype description, key words, gene names, NCBI IDs, and in‐house assigned IDs (Figure 5). In particular, we have generated a list of keywords describing phenotype features, such as “crinkled,” “mottled,” or “chlorotic” leaves. These keywords are listed on the web page, and the user can select one keyword from the list. Further, nucleotide sequences used for VIGS are provided in the database and can be bioinformatically analyzed for their silencing efficiency and for off‐target gene silencing using the VIGS tool (Fernandez‐Pozo, Rosli, Martin, & Mueller, 2015), siRNA scan (http://bioinfo2.noble.org/RNAiScan.htm), or pSSRNAit (http://plantgrn.noble.org/pssRNAit/) options integrated into the web site (Figure 5). The information derived from *N. benthamiana* can also be used to analyze several phylogenetically related crop plants, a large number of them listed in earlier literature (Becker & Lange, 2010). Further, results obtained based on information from *N. benthamiana* can be extended to other plants. For example, functional relevance of several genes identified from peanut was demonstrated in *N. benthamiana* under drought using VIGS (Senthil‐Kumar, Govind, Kang, Mysore, & Udayakumar, 2007). Considering the large number of genes and their silenced phenotypes available in the database, potential biological function of a bioinformatically predicted gene can be identified. Many plants did not show a visible phenotype upon silencing, and this could be due to inefficiency in gene silencing. Further, redundancy in the function of many genes and plasticity in metabolic pathways are other reasons for the absence of visible phenotype in some of the silenced plants. In contrary, some of the gene silencing phenotypes reported in the database could be due to off‐target silencing. Therefore, researchers should do further validation of the gene‐silenced phenotype. This database only provides a starting point for gene functional analyses. Researchers working with organisms other than plants can also use the database for the functional relevance of the orthologous genes of their species of interest. For example, heat‐shock protein 70 (HSP70, AJ001365) from *Drosophila auraria* nucleotide sequence BLAST in VIGS database showed five hits matching to HSP70 proteins. One of the top hits matching to *N. benthamiana* gene enlisted in the database was *NbHSP70* (NbTI07E09). Sequence annotation information with GO terms clearly indicated the putative function of this protein in the plant. Further, the gene‐silenced plants were stunted and showed pale yellow leaves along with spotted cell death and similar phenotype was previously reported *NbHSP70* in silenced plants (Kanzaki et al., 2003; Senthil‐Kumar, Govind, et al., 2007). This suggests that NbHSP70 could be involved in basal metabolic process that is required to maintain growth and normal cellular activities of a plant. Indeed, HSP70 has been shown to be an important protein needed for the cellular function in many plants and animals (Mayer & Bukau, 2005).

Unique features of the VPGD are as follows: (i) provides ready access for gene to phenotype information; (ii) has the potential to provide functional relevance of genes from over 20 species of *Solanaceae* family; (iii) provides multiple uses for gene sequence information, namely functional annotation (GO terms), phenotype description upon gene silencing, and VIGS construct for a particular gene sequence; (iv) has three input options namely gene ID/NCBI ID, sequence, and phenotype key words to search for user‐desired information; (v) has built‐in BLAST search along with detailed display of results; (vi) provides access to additional tools such as off‐target prediction, VIGS tool, and link to sequences/GO terms; and (vii) indicates availability of gene silencing construct.

## 5 CONCLUSIONS

The VIGS phenomics and functional genomics database (VPGD) is a unique resource that hosts large‐scale phenotypic information. Specifically, the database provides one‐stop access to genotype‐to‐phenotype information for over one thousand genes in *N. benthamiana* and closely related plant species. VIGS is a robust method for generating phenomic data for a large number of genes in a short time span. VPGD can be a model to develop phenomics database for other plant species. The aim of VPGD was to provide information on putative gene function and silencing phenotypes, without performing an experiment, to a wide range of plant species within Solanaceae family. VPGD provides putative gene function information for a large number of genes for plant species that have limited or no genetic resources (e.g., mutant collection).

## CONFLICT OF INTERESTS

Authors declare that they have no competing interest.

## AUTHOR CONTRIBUTIONS

KSM and MS‐K framed the concept and coordinated the project. MS‐K and HKL performed VIGS screen and other experiments and recorded the data. MS‐K and VD sequenced the cDNA libraries. OP, YL, GBM, and SPD‐K developed cDNA libraries. MS‐K, CMR, PX, JVE, SC, and KW contributed to phenotype pictures and VIGS screen. MS‐K compiled the EST and phenotype data. MS‐K, MW, and VR annotated EST sequences and phenotype information. MS‐K and KSM provided the outline. MW and JC developed the web site. MS‐K wrote the manuscript, and KSM and GBM edited it. All authors read and commented on the manuscript.
